# Multicolor Light-Induced Immune Activation via Polymer Photocaged Cytokines

**DOI:** 10.1021/acs.biomac.2c01207

**Published:** 2023-02-06

**Authors:** Lacey A. Birnbaum, Emily C. Sullivan, Priscilla Do, Biaggio Uricoli, Sunil S. Raikar, Christopher C. Porter, Curtis J. Henry, Erik C. Dreaden

**Affiliations:** † Coulter Department of Biomedical Engineering, Georgia Institute of Technology and Emory University, Atlanta, Georgia 30332, United States; ‡ Molecular and Systems Pharmacology Graduate Program, Emory University School of Medicine, Atlanta, Georgia 30307, United States; § Winship Cancer Institute of Emory University, Atlanta, Georgia 30322, United States; ∥ Department of Pediatrics, Emory School of Medicine, Atlanta, Georgia 30322, United States; ⊥ Aflac Cancer and Blood Disorders Center of Children’s Healthcare of Atlanta, Atlanta, Georgia 30322, United States; # Petit Institute for Bioengineering and Bioscience, Georgia Institute of Technology, Atlanta, Georgia 30322, United States

## Abstract

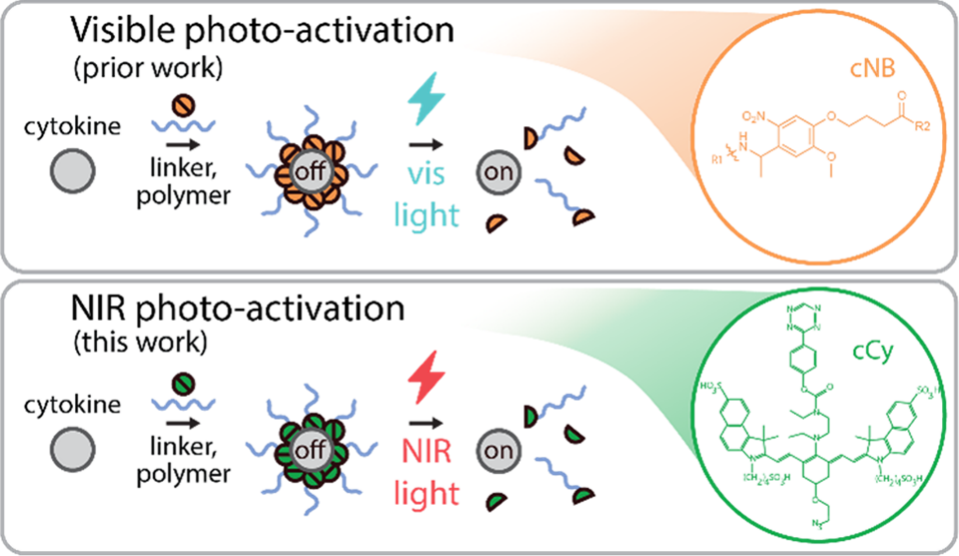

Cytokines act as potent, extracellular signals of the human immune system and can elicit striking treatment responses in patients with autoimmune disease, tissue damage, and cancer. Yet, despite their therapeutic potential, recombinant cytokine-mediated immune responses remain difficult to control as their administration is often systemic, whereas their intended sites of action are localized. To address the challenge of spatially and temporally constraining cytokine signals, we recently devised a strategy whereby recombinant cytokines are reversibly inactivated via chemical modification with photo-labile polymers that respond to visible LED light. Extending this approach to enable both in vivo and multicolor immune activation, here we describe a strategy whereby cytokines appended with heptamethine cyanine-polyethylene glycol are selectively re-activated ex vivo using tissue-penetrating near-infrared (NIR) light. We show that NIR LED light illumination of caged, pro-inflammatory cytokines restores cognate receptor signaling and potentiates the activity of T cell-engager cancer immunotherapies ex vivo. Using combinations of visible- and NIR-responsive cytokines, we further demonstrate multiwavelength optical control of T cell cytolysis ex vivo, as well as the ability to perform Boolean logic using multicolored light and orthogonally photocaged cytokine pairs as inputs and T cell activity as outputs. Together, this work demonstrates a novel approach to control extracellular immune cell signals using light, a strategy that in the future may improve our understanding of and ability to treat cancer and other diseases.

## Introduction

Cytokines orchestrate a range of biological processes and act as key signaling molecules of the human immune system, which regulate inflammation as well as immune cell migration, proliferation, and differentiation.^1−4^ Yet, despite their central importance to immune homeostasis, the clinical approval and use of recombinant cytokine immunotherapies to date have been hampered in part due to the pleiotropic effects of these molecules and their relatively low cell- or tissue-specificity that leads to dose-limiting or off-target side effects.^5^ For example, cardiopulmonary toxicities were observed in early clinical studies of aldesleukin (rIL-2) immunotherapy in cancer, and poor treatment outcomes were associated with the drug-induced expansion of immunosuppressive Tregs.^6^ Likewise, early trials of rIL-12 and rIL-15 immunotherapy were limited by significant hematologic and hepatic toxicity, as well as macrophage activation syndrome and intense cytokine secretion, respectively.^7,8^

Given their potent induction of antitumor immunity, various approaches to achieve cytokine cell- or tissue-specificity have been employed to date and include cytokine secretion by engineered cells, as well as affinity targeting, polymer conjugation, protein mutation, and de novo protein design.^9,10^ The use of various stimuli to locally activate cytokine prodrugs represents another promising approach to mitigate the off-target effects of cytokines and includes approaches to activate cytokine fusion proteins^11−13^ and drug carriers^14,15^ via proteolysis as well as those that release cytokines from protein-crosslinked nanoparticles in response to reducing conditions at the T cell surface.^16,17^

To address the challenge of improving the cell- and tissue-specificity of recombinant cytokines, we recently developed a strategy whereby cytokine activity (e.g., IL-2, IL-12, and IL-15) can be blunted via conjugation with photo-labile polyethylene glycol (PEG) and recovered via brief, in this case blue, LED light exposure.^18^ While these studies demonstrated the feasibility of in vivo cytokine photoactivation using tissue phantoms, we concluded that the clinical applications of this approach were limited to cutaneous or subdermal tissues and those accessible by in situ light sources (e.g., catheter-guided fiber lasers) as a result of efficient absorption and scattering of light by tissues and biological fluids at visible wavelengths. In contrast to visible light, near-infrared (NIR) photons can more efficiently penetrate human tissues including bone, skin, and muscle up to several centimeters,^19^ and diagnostic methods relying on NIR light sources have demonstrated clinical utility in optical coherence tomography and NIR spectroscopy applications.

Leveraging the ability of NIR photons to efficiently penetrate human tissues, here we advance our prior approach^18^ to reversibly photocage immunostimulatory cytokines by using photo-labile PEG that de-shields in response to NIR rather than visible light. We show that LED light exposure of NIR-photocaged cytokines can be used to reversibly modulate cognate cytokine receptor signaling and enhance the activity of bispecific cancer immunotherapies that rely on T cell-dependent cytolysis. Using combinations of visible and NIR light-responsive cytokines, we further demonstrate orthogonal, multicolor photocontrol of T cell cytolytic activity ex vivo and the novel ability to perform Boolean logic using multicolor light and differentially photocaged cytokines. This work expands the working toolbox for protein photocaging and may be extended in the future to a range of other biological systems that rely on extracellular signaling or synthetic cytokine/receptor pairs.

## Methods

### Materials and Supplies

Unless otherwise specified, reagents were used as received without further purification. Recombinant human IL-2 (200-02, Peprotech), recombinant human IL-15 (570308, Biolegend), recombinant mouse scIL-12 (130-096, Miltenyi Biotec), and recombinant human IL-12 (CT050-HNAH, Sino Biological). Sulfo-Cyanine7 NHS ester (Lumiprobe), DBCO-NHS ester (1160, Click Chemistry Tools), NHS-PEG-TCO ester (A137-10, Click Chemistry Tools), poly(ethylene glycol)methyl ether DBCO (20 kDa, A120-100, Click Chemistry Tools), and poly(ethylene glycol)methyl ether azide (20 kDa, Nanocs). Polyacrylamide gels (Bio-Rad, 4–16 wt %). IFNγ ELISA (430104, Biolegend). CD19 antibody (BD Biosciences Clone SJ25C1) and PD-L1 antibody (eBioscience Clone MIH1).

### Cell Lines and Primary Cells

HEK-Blue IL-12 cells (Invivogen) were cultured according to the manufacturer’s recommendations in Dulbecco’s modified Eagle’s medium (Corning) supplemented with 10% heat-inactivated fetal bovine serum (FBS) (VWR) and 100 IU/mL pen-strep (VWR). NALM-6 cells (gifted from Dr. Lia Gore, University of Colorado) were cultured in RPMI 1640 supplemented with 10% FBS and 100 U/mL penicillin and 100 μg/mL streptomycin. Primary CD8+ T cells were obtained from normal donor human buffy coats (LifeSouth) via Ficoll-Paque (Cytiva) gradient selection for peripheral blood mononuclear cells followed by human CD8+ T cell negative magnetic selection (Stem Cell Technologies). Primary CD8+ T cells were thawed in supplemented RPMI 1640 2–12 h prior to the start of experiments.

### cCy Synthesis

2-((*E*)-2-((*E*)-2-((2-(((4-(1,2,4,5-Tetrazin-3-yl)phenoxy)carbonyl)(ethyl)amino)ethyl)(ethyl)amino)-5-(2-azidoethoxy)-3-((*E*)-2-(1,1-dimethyl-7-sulfo-3-(4-sulfobutyl)-1,3-dihydro-2*H*-benzo[*e*]indol-2-ylidene)ethylidene)cyclohex-1-en-1-yl)vinyl)-1,1-dimethyl-7-sulfo-3-(4-sulfobutyl)-1*H*-benzo[*e*]indol-3-ium, cCy, was prepared at-scale by eMolecules (San Diego, CA) from 1,4-dioxaspiro[4.5]decan-8-one and purified to >96% via preparatory high-performance liquid chromatography. cCy was structurally characterized by ^1^H NMR, ^13^C NMR, and UPLC–MS using an Acquity BEH C-18 column (1.7 μm): buffer A, 5 mM ammonium acetate in water; buffer B, acetonitrile; gradient was 10–90% A over 6 min at a 0.3 mL/min flow rate. See Supplementary Information for additional details.

### cCy Spectroscopic Characterization

cCy was reconstituted to 50 mM in dry dimethyl sulfoxide (Thermo Fisher Scientific) and further diluted 1000× in 1× phosphate-buffered saline (PBS) (Corning) for characterization studies. Absorption and emission spectra were measured on a Spectramax iD3 plate reader (Molecular Devices) at 10 nm increments. Photolysis kinetics were monitored using a NanoDrop One spectrophotometer following irradiation with a 785 nm diode laser (Crysta, 110 mW/cm^2^). Data were fitted using a one-phase decay model in Graphpad Prism.

### Photolithographic Patterning

1 mm silicone spacers with 9 mm diameter circular openings (Electron Microscopy Sciences) were adhered to amine-coated glass slides (Nanocs). The slides were functionalized with NHS-TCO at a 10:1 NHS/amine ratio overnight at RT. Wells were then washed 6× with deionized water, 3× with 0.2% sodium dodecyl sulfate (SDS), and again 3× with deionized water, followed by conjugation with cCy at a 3:1 cCy/amine ratio overnight at RT in tris buffered saline with 0.1% Tween (TBST). Wells were then washed 5× with TBST (0.1% Tween 20) and covered in 50% glycerol. A chrome-coated quartz photomask was then placed on the silicone isolator, and a collimated 730 nm LED (ThorLabs) was used to irradiate the slide for 30 min (100 mW/cm^2^). The irradiated slide was then washed 5× with TBST and mounted with Prolong Diamond antifade media (Life Technologies) for imaging. Patterned slides were imaged on an Odyssey CLX fluorescence imaging scanner. Image features were quantified and normalized using ImageJ.

### Conjugation with cCy-PEG

Cytokines were modified with cCy-PEG using carbodiimide and TCO–tetrazine chemistry, respectively, using methods adapted from Perdue et al.^18^ Briefly, rIL-12 was sequentially reacted overnight with a 10–120-fold molar excess of NHS-TCO followed by a 40–480-fold molar excess of cCy and a 160–1920-fold molar excess of DBCO-mPEG_20kDa_ (4 °C with 800 rpm rotatory agitation). Excess reactants were removed via 7 kDa size exclusion chromatography (Zeba Spin, Thermo Fisher Scientific) in some experiments. IL-15-cCy-PEG was prepared using methods identical to IL-12-cCy-PEG except for the use of a 10–20-fold molar excess of NHS-TCO, a 40–80-fold molar excess of cCy, and a 160–320-fold molar excess of DBCO-mPEG_20kDa_ (see the Supplementary Information).

### Conjugation with cNB-PEG

rIL-2 was modified with cNB-PEG using carbodiimide and azide–alkyne chemistry, respectively, using methods described in Perdue et al.^18^ Briefly, rIL-2 was diluted in 150 mM sodium phosphate buffer (pH 8.5) containing 0.5 mM SDS and sequentially reacted with an 80-fold molar excess of cNB, followed by N_3_-mPEG_20kDa_ at a 400-fold molar excess in PBS (4 °C with 800 rpm rotatory agitation, overnight).

### Photocaged Cytokine Characterization

Protein electrophoretic mobility was characterized via polyacrylamide gel electrophoresis (4–16% weight) under reducing conditions. Protein bands were stained with Coomassie G250 (Bio-Rad) and visualized using a Licor CLx imager. Coomassie was imaged in the 700 channel of the imager, while cCy was imaged in the 800 channel.

### Cytokine Photoactivation

Irradiation of IL-12-cCy-PEG was performed in transparent film-covered U-bottom 96-well plates following illumination with a 730 nm LED (ThorLabs, 100 mW/cm^2^, 30 min). cCy-PEG conjugates were diluted to 0.125–0.15 μg/mL in HEPES-buffered saline (Invitrogen, pH 7.4) containing 0.1% bovine serum albumin and 0.02% Tween 20 prior to irradiation. Irradiation of IL-2-cNB-PEG was similarly performed except for the use of a 365 nm LED (ThorLabs, 40 mW/cm^2^, 20 min) and pre-dilution to 0.15 μg/mL in PBS (pH 7.4). Illumination was performed in all cases at 4 °C.

### Cytokine Receptor Signaling Assay

HEK-293 SEAP reporter cells (Invivogen) were plated at 5 × 10^4^ cells per well in complete growth media within 96-well plates or at 2.5 × 10^4^ cells per well in a 384-well plate and treated with equimolar amounts of rh or caged cytokine, with and without light exposure. After 24 h, 20 μL of the supernatant was withdrawn per well, diluted 10-fold in Quanti-Blue reagent (rep-qbs, Invivogen), and placed at 37 °C for 0.25–3.0 h before reading absorbance at 620 nm (Spectramax iD3 plate reader). Dose–response curves were calculated using Graphpad Prism with a four-parameter fit and normalized to background and signal from unmodified proteins.

### T Cell Cytolysis Assays

CD19+ NALM-6 cells (B cell acute lymphoblastic leukemia) were stained with CellTrace Violet (Thermo Fisher Scientific), and primary human CD8+ T cells were stained with either CellTrace Yellow (Thermo Fisher Scientific) or CFSE (Tonbo Biosciences) for ≥1 h prior to co-culture. During co-culture, CD8+ T cells and 5 × 10^4^ NALM-6 cells were plated with 0.5–1 ng/mL of blinatumomab (Invivogen) and cytokine, if applicable, in a U-bottom 96-well plate for 72 h (1:1 or 1:2 E:T, as indicated). Cytokine concentrations were in the ranges of 5–1000 IU/mL (IL-2) and 7–400 ng/mL (IL-12). After 72 h, cells were stained with the Near-IR Live/Dead stain (Thermo Fisher Scientific) for viability and immediately assessed by flow cytometry. Specific lysis was calculated as % Specific Lysis = 100 × [(Treated CTV + LD+) – (Untreated CTV + LD+)]/(100 – Untreated CTV + LD+), where LD is the viability stain, CTV is CellTrace Violet, and the untreated condition received no blinatumomab. The supernatant from these cultures was immediately frozen at −80 ° C. For IFNγ ELISA (Biolegend), co-culture supernatants were diluted 10–100-fold and assayed according to the manufacturer’s instructions. During logic gate experiments, a positive output was based upon inter-donor variability and defined as cytolysis within 50 % CI of values obtained from combined treatment with rIL-12, rIL-2, and blinatumomab. To assess changes in the tumor cell phenotype, cells were stained for PD-L1 and CD19 for 1 h at a 1:100 antibody dilution, washed, and immediately assessed by flow cytometry.

### Statistical Analysis

Unless otherwise noted, all *p*-values were calculated using either one-way analysis of variance with Tukey post hoc correction or one-way *T* test (Graphpad Prism), depending on the number of experimental conditions.

## Results and Discussion

**Figure 1:**
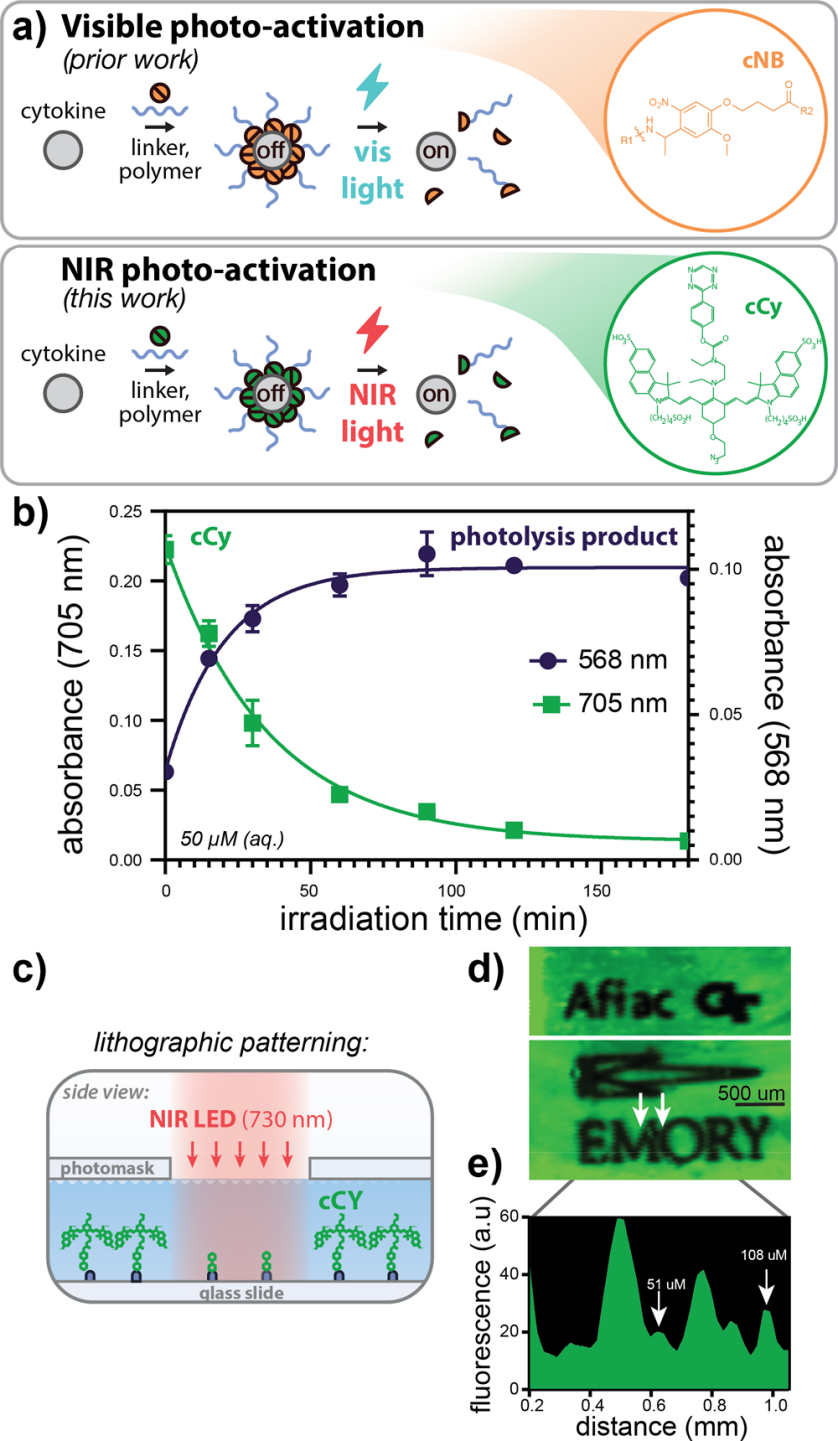
NIR photo-labile linker, cCy, undergoes rapid, spatially well resolved photocleavage. (a) Strategy for blue and NIR photon-gated protein activation via cCy-linked polymer conjugation. (b) Aqueous NIR photolysis kinetics of cCy as measured by UV–vis spectroscopy of parent (blue) and decomposition (purple) products during irradiation (785 nm, 110 mW/cm^2^). (c) Schematic illustrating cCy immobilization to TCO-functionalized glass slides and subsequent aqueous-phase photolithographic patterning (730 nm, 100 mW/cm^2^). (d) Fluorescence micrographs of cCy (green) photopatterns and (e) corresponding image line scans demonstrating approximately 51 μm lateral resolution (785 nm ex, 812–832 nm em). Data in (b) represent the mean ± SD of three technical replicates per time point.

To achieve NIR control of cytokine signaling, we first devised a heterobifunctional linker that would undergo photolysis to liberate one of two conjugation sites upon light exposure. Gorka et al. previously showed that C4′-dialkylamine-substituted heptamethine cyanine fluorophores undergo regioselective photooxidation followed by C4′–N hydrolysis and cyclization to liberate aromatic alcohols following NIR light exposure.^20^ We surmised (i) that substitution of this leaving group with a phenolic tetrazine would enable the photo-induced loss of a variety of compounds appended via *trans*-cyclooctene (TCO) click chemistry and (ii) that the addition of a distal azido ethoxy group to the backbone cyclohexene would enable the linker to be protein-anchored via orthogonal azide–alkyne click chemistry.^21−23^ As anticipated, the subsequent heterobifunctional linker (hereafter cCy) exhibited strong NIR absorption and fluorescence (ex/em, 705/810 nm, Figures S1 and S2, as well as rapid photolysis as characterized via UV–vis absorption spectroscopy following NIR irradiation (785 nm, photooxidation *t*_1/2_ 25 min; Figures 1a,b and S3). Notably, the spectral blue-shift that we observe here following photo-irradiation strongly resembles that reported by Gorka et al., who found that heptamethine cyanine photolysis occurs initially via cleavage of the pi-conjugated dye backbone.^20^ We hypothesize that the photolysis of cCy here is mechanistically similar. To characterize the spatial resolution in which cCy can be photocleaved, we next immobilized it to TCO-functionalized glass slides via click chemistry and exposed these slides to collimated NIR LED light (730 nm) under aqueous conditions through a custom lithographic mask (Figure 1c). Using intrinsic fluorescence of cCy, we observed a lateral resolution for photocleavage of ∼51 μm via epifluorescence imaging (Figure 1c–e), a finding that is significant in that this dimension approaches that of single mammalian cells, thus demonstrating its potential utility in intravital imaging studies,^24−26^ as well as ex vivo or in vitro culture studies in the future.^27,28^

**Figure 2:**
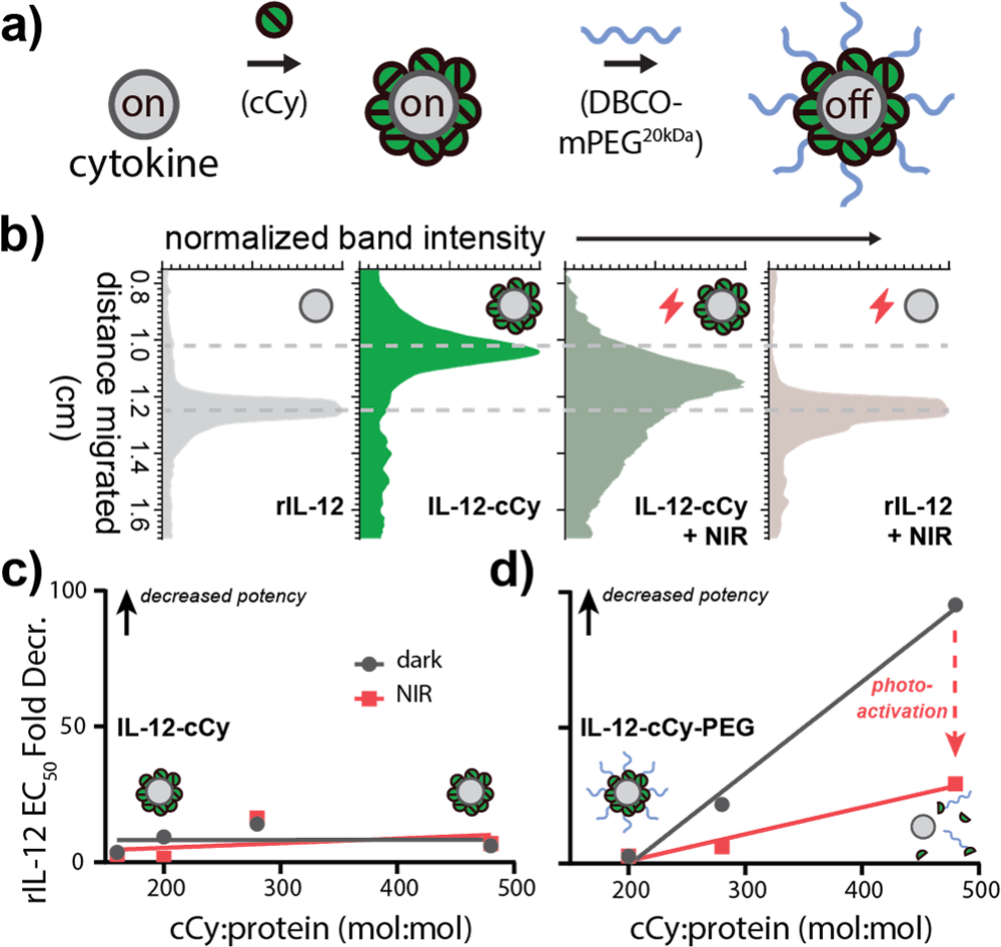
NIR light exposure of IL-12-cCy-PEG restores the protein size and de-represses pSTAT4 signaling. (a) Schematic illustrating the modification of recombinant IL-12 with cCy-PEG_20kDa_. (b) Densitometry of IL-12 and IL-12-cCy with and without NIR light exposure illustrating a decrease then recovery in electrophoretic mobility following cCy conjugation and subsequent NIR light exposure, respectively (730 nm, 100 mW/cm^2^). IL-12 dose-dependent STAT4 activation (EC_50_) with varying (c) cCy and (d) cCy-PEG conjugation ratio as measured via the SEAP/chromogenic assay using STAT4 reporter cells (24 h) with and without NIR light exposure (730 nm, 100 mW/cm^2^) and normalized as fold-change relative to rIL-12 EC_50_. Data in (c) and (d) represent the mean ± SD of three technical replicates.

Having demonstrated that cCy undergoes rapid and spatially well defined photolysis, we next investigated whether the conjugation of pro-inflammatory cytokines with cCy-PEG modulates their activity on T cells. Using carbodiimide and TCO–tetrazine chemistry, we modified surface primary amines of recombinant IL-12 to yield proteins conjugated with either cCy or cCy-PEG_20kDa_ (Figure S4), and, using reporter cells that secrete alkaline phosphatase in a pSTAT4-dependent manner, we identified cCy conjugation densities that increase the apparent molecular weight of rIL-12 while preserving its signaling activity (Figure 2a–c). Subsequent NIR LED light exposure of IL-12-cCy led to near-complete restoration of IL-12 electrophoretic mobility. After confirming that high-density cCy conjugation failed to significantly diminish IL-12 activity, we next conjugated IL-12 with cCy-PEG, observing a 95 ± 6-fold decrease in potency relative to rIL-12 as measured by pSTAT4 reporter cell activity (Figure 2c,d). Following NIR photolysis, we observed a 66 ± 7-fold recovery in IL-12 potency (EC_50,24h_) consistent with prior findings that PEG can sterically shield receptor binding interactions between proteins and their cognate receptors in a PEG density- and molecular weight-dependent manner.^18^ To confirm the generalizability of this approach to photocaging, we demonstrated a comparable increase and recovery in rIL-15 electrophoretic mobility following conjugation with cCy-PEG and NIR light exposure (Figure S5). Together, these data demonstrate that cCy-PEG reversibly modulates the size and ex vivo activity of rIL-12 on T cells in an NIR photon-dependent manner.

**Figure 3:**
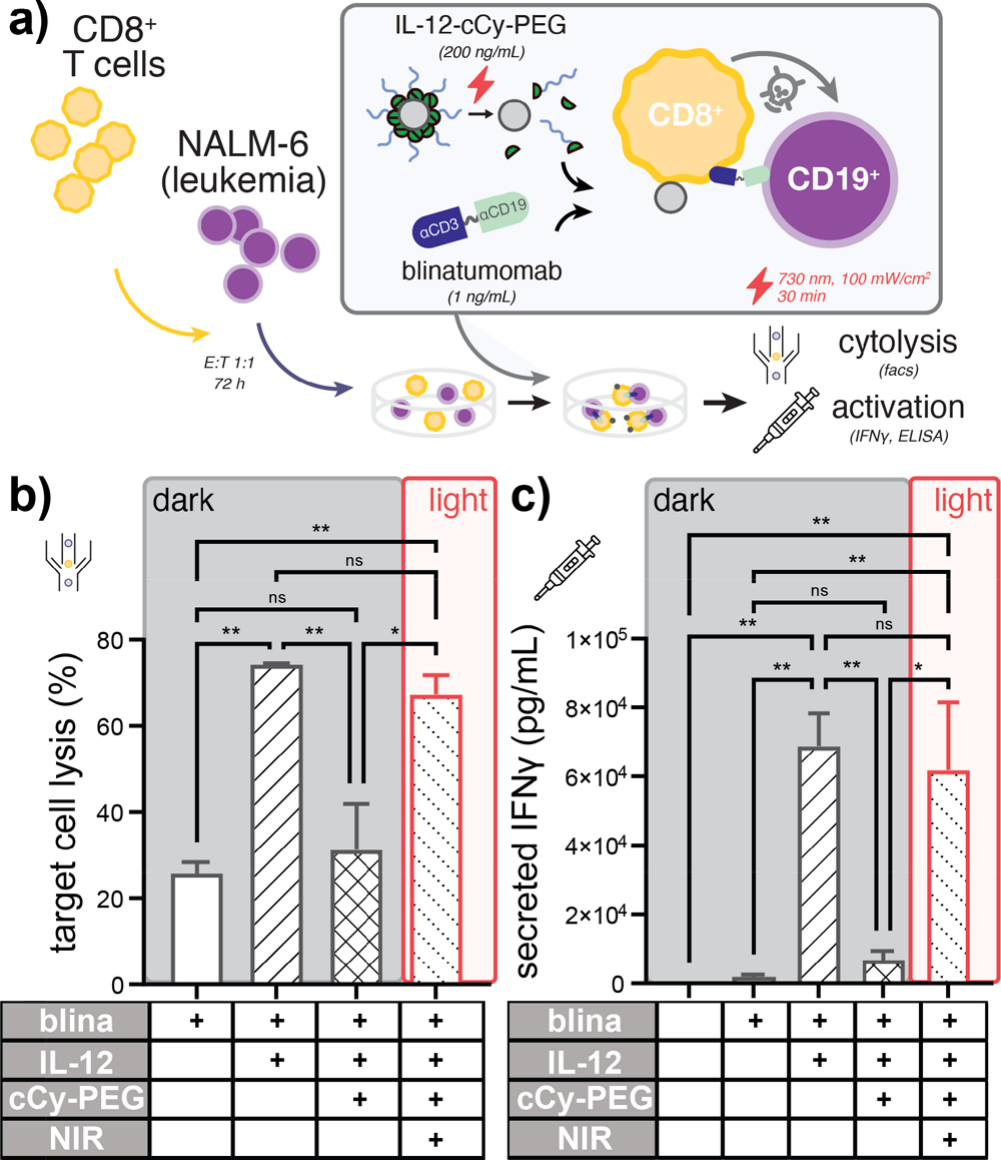
NIR light modulates the potency of blinatumomab immunotherapy when co-treated with IL-12-cCy-PEG. (a) Schematic illustrating assay conditions in which co-cultures of labeled primary human CD8+ T cells and labeled CD19+ NALM-6 B-ALL cells are treated with blinatumomab and rIL-12 or IL-12-cCy-PEG, with and without NIR light illumination. (b) CD19-specific target cell lysis demonstrating the loss and recovery of blinatumomab cytolytic activity following cCy-PEG photocaging and subsequent NIR LED illumination (730 nm), respectively, as measured by flow cytometry. (c) IFNγ measurements indicating repression and de-repression of IL-12-dependent T cell activation following polymer photocaging and NIR light exposure, respectively, as measured by ELISA of co-culture supernatants. Data in (b) and (c) represent the mean ± SD of at least two technical replicates from a representative healthy T cell donor. +IL12 indicates that the protein is present, +cCy-PEG indicates caging and +NIR indicates light exposure inducing uncaging.

In prior work, we demonstrated that recombinant IL-12 can enhance the cytolytic activity of the T cell bispecific immunotherapy, blinatumomab, currently approved to treat patients with relapsed and refractory B cell acute lymphoblastic leukemia (B-ALL).^29,30^ To determine whether IL-12-cCy-PEG can likewise improve blinatumomab-induced cytolysis in an NIR light-dependent manner, we treated co-cultures of labeled primary human CD8+ T cells and CD19+ NALM-6 B-ALL cells with blinatumomab and rIL-12 or IL-12-cCy-PEG, with and without NIR light exposure, and measured CD19-specific cell lysis and T cell activation via flow cytometry and IFNγ ELISA, respectively (Figure 3a–c). As anticipated, we observed no significant change in blinatumomab-induced cytolysis or T cell activation following co-culture with IL-12-cCy-PEG without illumination; however, following NIR LED exposure of the protein, we observed an increase in both CD19-specific lysis and IFNγ secretion that was statistically indistinguishable from that enhanced by rIL-12.

**Figure 4:**
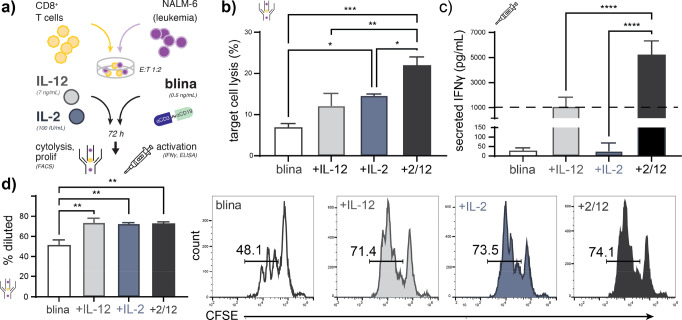
Unmodified IL-12 and IL-2 cooperatively enhance blinatumomab-induced cytolytic activity and CD8 T cell activation ex vivo. (a) Schematic illustrating assay conditions in which co-cultures of labeled primary human CD8+ T cells and labeled CD19+ NALM-6 B-ALL cells are treated with blinatumomab and wild-type rIL-12 or rIL-2. (b) Blinatumomab-induced target cell lysis illustrating improved cytolytic activity from IL-12 or IL-2 co-treatment, as well as additive cooperative effects from their combination as measured by flow cytometry. Parallel measurements of (c) IFNγ production and (d) T cell proliferation demonstrating that wild-type rIL-12 and rIL-2 synergize to enhance CD8+ T cell activation but only sub-additively impact T cell proliferation as measured by ELISA of co-culture supernatants and flow cytometric dye dilution, respectively. The dashed line in (c) denotes the additive expectation as assessed via the response additivity model. Data in (b)–(d) represent the mean ± SD of at least two technical replicates from a representative healthy T cell donor.

After demonstrating that NIR photocaging can be used to modulate the influence of IL-12 on blinatumomab-induced cytolysis, we asked whether other cytokines might cooperate with IL-12 to further improve drug activity. Prior studies demonstrated that (i) IL-2 treatment upregulates IL-12R on T cells, (ii) IL-12 treatment upregulates IL-2R (CD25) on CD8+ T cells, and (iii) the combination of both IL-12 and IL-2 synergizes to improve tumor control in syngenetc mouse models of renal cell carcinoma.^31−33^ To determine whether cooperativity between IL-12 and IL-2 can further enhance blinatumomab activity ex vivo, we co-cultured CFSE-labeled primary human CD8+ T cells and CellTrace Violet-labeled CD19+ NALM-6 B-ALL cells with blinatumomab in the presence or absence of each recombinant cytokine or their combination (Figure 4a). We observed that rIL-2 improved drug-induced cytolysis to levels comparable with rIL-12 alone and that both proteins additively combined with blinatumomab to augment CD19-specific cell lysis as measured by flow cytometry (Figure 4b), despite that neither blinatumomab nor cytokine treatment impacted target CD19 or PD-L1 immune checkpoint ligand expression on leukemic B cells (Figure S6). Interestingly, while rIL-12 and rIL-2 combined only sub-additively to alter T cell proliferation, they synergized to enhance IFNγ secretion as measured by dye dilution and ELISA of co-culture supernatants, respectively (Figure 4c,d). Together, these data support that (i) recombinant IL-2 alone may be used to enhance the potency of blinatumomab immunotherapy and (ii) cytokine-enhanced T cell activation rather than T cell expansion is a dominant contributor to the combined impact of IL-12 and IL-2 on blinatumomab activity observed here.

**Figure 5:**
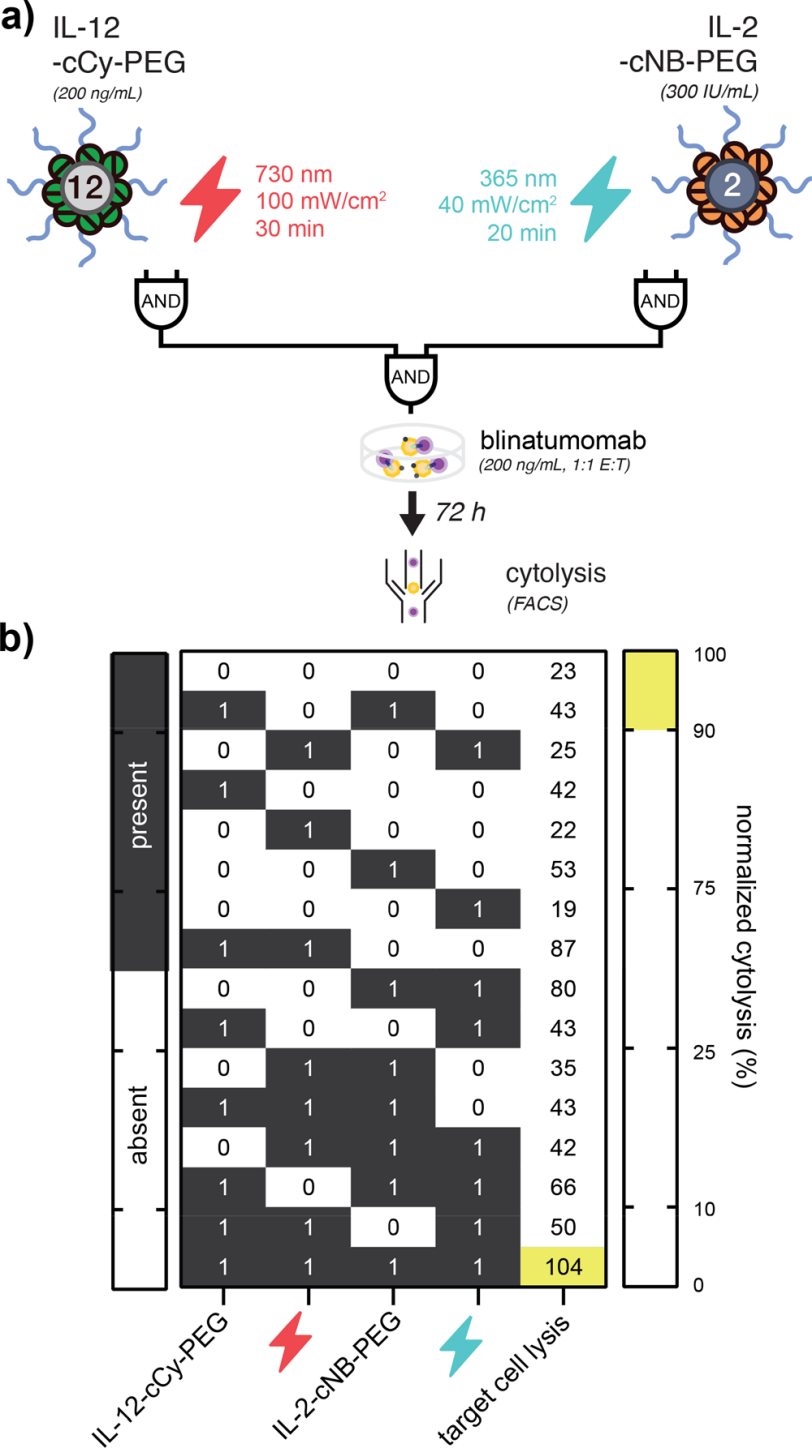
Combined photonic and biological circuit based upon orthogonally photocaged IL-12 and IL-2. (a) Schematic illustrating an AND–AND logic gate whereby (i) blue and NIR LED light as well as IL-2-cNB-PEG and IL-12-cCy-PEG represent digitized inputs and (ii) normalized blinatumomab-induced target cell lysis represents a digitized output. (b) Truth table representing all possible combinations of initial states (green) and true outputs (blue), overlaid with observed output values of normalized drug-induced cytolysis as measured via flow cytometry. Data in (b) represent the mean of two–four technical replicates from two healthy T cell donors.

Having demonstrated that rIL-12 and rIL-2 cooperate to improve blinatumomab-induced cytolysis, we next asked whether the conjugation of each protein with wavelength-orthogonal photocages could be used to control their combined effect on CD8+ T cells, blinatumomab, and CD19+ leukemic B cells via multicolor LED light. In prior work, we developed a strategy to photocage IL-2 with blue LED light-responsive photocages based upon *o*-nitrobenzyl-linked PEG_20kDa_ (i.e., IL-2-cNB-PEG).^18^ Using this conjugate and the NIR-responsive IL-12 conjugate described here (IL-12-cCy-PEG), we constructed a Boolean circuit that combines AND gates in which we digitize the presence or absence of NIR or visible LED light, as well as the presence or absence of discrete concentrations of IL-12-cCy-PEG or IL-2-cNB-PEG (Figures 5a and S7). Using these components as inputs and blinatumomab-induced cytolysis level as a digitized output, we observed full concordance with the expected AND–AND gate truth table whereby above-threshold B cell lysis was observed only in the presence of both caged cytokines, as well as both visible and NIR LED light (Figure 5b). Together, these data demonstrate that multicolor light can be used to interface with and modulate ex vivo immune responses when combined with photocaged cytokines.

Extending our prior approach to optically modulate cytokine signaling networks using visible LED light, here we describe a method (i) to photocontrol T cell cytokine signaling via tissue penetrant NIR light and (ii) to orthogonally modulate cooperative cytokine signaling via combined visible and NIR LED light. Using a photo-labile, heterobifunctional linker based upon heptamethine cyanine fluorophores (cCy), we demonstrated rapid NIR photolysis kinetics with a photooxidation half-life of ∼25 min, notably fast as compared with proteolytic activation methods that can exceed several hours in activation half-life. Using this novel photo-labile linker, we further observed a minimum lateral resolution for NIR photoactivation of ∼51 μm and anticipate further enhancements in resolution using focusing optics and/or multiphoton laser excitation (e.g., 1560 nm pulsed laser irradiation) as is common in high-resolution microscopy applications.^34^ Such approaches when utilized with the NIR photocaging strategy described here may find strong utility in intravital imaging applications,^24−26^ as well as ex vivo studies of organoids and embryo-like body development where high spatial control of protein delivery is necessary for proper cell organization.^27,28^

In prior work,^29,30^ we demonstrated that rIL-12 could potentiate the therapeutic effects of the T cell engager blinatumomab (Blincyto) ex vivo, and here we further demonstrate the NIR photocontrol of this enhanced activity via NIR illumination of cCy-PEG-conjugated rIL-12. This finding is significant as conventional rIL-12 therapies are well known to be associated with severe hematologic and hepatic toxicities that may potentially compound with those observed from blinatumomab when delivered in combination. Using the approach described here, we observed a >98% reduction in signaling EC_50_ that we anticipate would mitigate such adverse effects in vivo via sustained or localized (e.g., bone marrow) delivery via NIR activation.

The observation that rIL-2, like rIL-12, potentiates the therapeutic activity of blinatumomab is also significant as this is the first report to our knowledge that supports this effect from IL-2, as well as the first to demonstrate cooperativity between these two cytokines in enhancing the cytolytic activity of T cell bispecifics. Like IL-12, there are numerous IL-2-based clinical-stage drug candidates including protein fusions, PEG–cytokine conjugates, and mRNA.^9,35,36^ At the same time, there are now (four) clinically approved T cell bispecific immunotherapies that include both hematologic cancers and solid tumors.^37,38^ Thus, these findings may support the future clinical development of combined, or chimeric, T cell bispecific and cytokine cancer immunotherapies in the future.

Finally, to exploit cooperativity between IL-12 and IL-2 in enhancing the ex vivo activity of blinatumomab, we constructed a Boolean circuit in which only 1 of 16 possible initial states—comprising caged IL-12, caged IL-2, blue light, or NIR light—would correspond to above-threshold cytolytic activity. In the future, this approach may be used to orchestrate or tune complex cytokine signaling networks using cocktails of caged cytokines and time- and/or wavelength-modulated light sources.

The finding that soluble proteins such as cytokines may be manipulated using exogenous light is also significant considering that the overwhelming majority of optical tools available to synthetic biologists such as photon-gated ion channels^39^ and light-dependent protein dimerization^40^ necessitate the use of live cells whose presence may be limiting in some systems or applications.

As discussed above, one salient barrier to the clinical application of recombinant cytokine immunotherapies in cancer is their relatively small size (typically 12–70 kDa) and therefore rapid excretion that necessitates frequent and/or high-dose and complex treatment management.^41,42^ In prior work, the addition of just a single PEG molecule to GM-CSF or interferon alfa-2b was found to be sufficient to confer prolonged circulation and tissue-drug exposure, which in turn improves the therapeutic index.^43^ Here, we likewise anticipate that prolonged circulation from IL-12-cCy-PEG relative to rIL-12 may enable it to more effectively elicit antitumor immune responses both alone and in combination with T cell bispecifics, immune checkpoint inhibitors, chimeric antigen receptor T cells, and autologous gamma delta T cell^44^ or NK cell^45,46^ therapies in the future where it may potentiate improved treatment outcomes.

## Conclusions

In summary, here we demonstrate abiological control of cytokine signaling networks using a multicolor light and prodrug strategy whereby recombinant cytokines are chemically modified with photo-labile polymers that de-repress their activity in response to low-power LED light. Given recent advancements in the development of highly efficient and wavelength-variable photocages,^47−49^ we anticipate further extensions of this approach in the future that will expand wavelength multiplexing capabilities and further exploit the utility of high spatial and temporal control over immune cell activation, migration, and differentiation.

## Supporting Information Available

cCy chemical structure, absorption/emission spectrum, UPLC–MS, NMR analysis, photolysis kinetics, treatment-induced surface protein expression, IL-15 conjugation with cCy-PEG, and NIR photocleavage (PDF)

## References

[ref1] Wang X.; Lupardus P.; LaPorte S. L.; Garcia K. C. Structural Biology of Shared Cytokine Receptors. Annu. Rev. Immunol. 2009, 27, 29–60. 10.1146/annurev.immunol.24.021605.090616. 18817510 PMC3981547

[ref2] Grivennikov S. I.; Greten F. R.; Karin M. Immunity, Inflammation, and Cancer. Cell 2010, 140, 883–899. 10.1016/j.cell.2010.01.025. 20303878 PMC2866629

[ref3] Watowich S. S.; Hong W.; Socolovsky M.; Klingmuller U.; Stefan N.; Lodish H. F. Cytokine Receptor Signal Transduction and the Control of Hematopoietic Cell Development. Annu. Rev. Cell Dev. Biol. 1996, 12, 91–128. 10.1146/annurev.cellbio.12.1.91. 8970723

[ref4] Griffith J. W.; Sokol C. L.; Luster A. D. Chemokines and Chemokine Receptors: Positioning Cells for Host Defense and Immunity. Annu. Rev. Immunol. 2014, 32, 659–702. 10.1146/annurev-immunol-032713-120145. 24655300

[ref5] Baldo B. A. Side Effects of Cytokines Approved for Therapy. Drug Saf. 2014, 37, 921–943. 10.1007/s40264-014-0226-z. 25270293 PMC7101846

[ref6] Wei S.; Kryczek I.; Edwards R. P.; Zou L.; Szeliga W.; Banerjee M.; Cost M.; Cheng P.; Chang A.; Redman B.; Herberman R. B.; Zou W. Interleukin-2 Administration Alters the CD4+FOXP3+ T-Cell Pool and Tumor Trafficking in Patients with Ovarian Carcinoma. Cancer Res. 2007, 67, 7487–7494. 10.1158/0008-5472.CAN-07-0565. 17671219

[ref7] Eng V. M.; Car B. D.; Schnyder B.; Lorenz M.; Lugli S.; Aguet M.; Anderson T. D.; Ryffel B.; Quesniaux V. F. The stimulatory effects of interleukin (IL)-12 on hematopoiesis are antagonized by IL-12-induced interferon gamma in vivo. J. Exp. Med. 1995, 181, 1893–1898. 10.1084/jem.181.5.1893. 7722464 PMC2191982

[ref8] Conlon K. C.; Lugli E.; Welles H. C.; Rosenberg S. A.; Fojo A. T.; Morris J. C.; Fleisher T. A.; Dubois S. P.; Perera L. P.; Stewart D. M.; Goldman C. K.; Bryant B. R.; Decker J. M.; Chen J.; Worthy T. Y. A.; Figg W. D.; Peer C. J.; Sneller M. C.; Lane H. C.; Yovandich J. L.; Creekmore S. P.; Roederer M.; Waldmann T. A. Redistribution, Hyperproliferation, Activation of Natural Killer Cells and CD8 T Cells, and Cytokine Production During First-in-Human Clinical Trial of Recombinant Human Interleukin-15 in Patients With Cancer. J. Clin. Oncol. 2014, 33, 74–82. 10.1200/JCO.2014.57.3329. 25403209 PMC4268254

[ref9] Uricoli B.; Birnbaum L. A.; Do P.; Kelvin J. M.; Jain J.; Costanza E.; Chyong A.; Porter C. C.; Rafiq S.; Dreaden E. C. Engineered Cytokines for Cancer and Autoimmune Disease Immunotherapy. Adv. Healthcare Mater. 2021, 10, 2002214 10.1002/adhm.202002214. PMC8651077 33690997

[ref10] Bonati L.; Tang L. Cytokine engineering for targeted cancer immunotherapy. Curr. Opin. Chem. Biol. 2021, 62, 43–52. 10.1016/j.cbpa.2021.01.007. 33684633

[ref11] Skrombolas D.; Sullivan M.; Frelinger J. G. Development of an Interleukin-12 Fusion Protein That Is Activated by Cleavage with Matrix Metalloproteinase 9. J. Interferon Cytokine Res. 2019, 39, 233–245. 10.1089/jir.2018.0129. 30848689 PMC6479256

[ref12] Bishop J. L.; Blackler R.; Volkers G.; Poffenberger M.; Yu I.; Smith J.; Khodabandehloo A.; Arrafi S.; Lau D.; Stangle L.; Stenberg L.; Zwierzchowski P.; Dude I.; Douda D.; Wickman G.; Proctor J.; Rowse G.; Madera L.; Desjardins G.; Afacan N.; Barnscher S.; Mills D.; Spreter T.; Dixit S. Abstract 1788: Increasing the therapeutic index of IL12 by engineering for tumor specific protease activation. Cancer Res. 2021, 81, 1788–1788. 10.1158/1538-7445.AM2021-1788.33483371

[ref13] Mansurov A.; Hosseinchi P.; Chang K.; Lauterbach A. L.; Gray L. T.; Alpar A. T.; Budina E.; Slezak A. J.; Kang S.; Cao S.; Solanki A.; Gomes S.; Williford J.-M.; Swartz M. A.; Mendoza J. L.; Ishihara J.; Hubbell J. A. Masking the immunotoxicity of interleukin-12 by fusing it with a domain of its receptor via a tumour-protease-cleavable linker. Nat. Biomed. Eng. 2022, 6, 819–829. 10.1038/s41551-022-00888-0. 35534574 PMC11155269

[ref14] Wang H.; Hou Y.; Hu Y.; Dou J.; Shen Y.; Wang Y.; Lu H. Enzyme-Activatable Interferon–Poly(α-amino acid) Conjugates for Tumor Microenvironment Potentiation. Biomacromolecules 2019, 20, 3000–3008. 10.1021/acs.biomac.9b00560. 31310511

[ref15] Park E.; Hart M. L.; Rolauffs B.; Stegemann J. P.; Annamalai T. Bioresponsive microspheres for on-demand delivery of anti-inflammatory cytokines for articular cartilage repair. J. Biomed. Mater. Res. 2020, 108, 722–733. 10.1002/jbm.a.36852. PMC6995991 31788947

[ref16] Tang L.; Zheng Y.; Melo M. B.; Mabardi L.; Castaño A. P.; Xie Y.-Q.; Li N.; Kudchodkar S. B.; Wong H. C.; Jeng E. K.; Maus M. V.; Irvine D. J. Enhancing T cell therapy through TCR-signaling-responsive nanoparticle drug delivery. Nat. Biotechnol. 2018, 36, 707–716. 10.1038/nbt.4181. 29985479 PMC6078803

[ref17] Eskandari S. K.; Sulkaj I.; Melo M. B.; Li N.; Allos H.; Alhaddad J. B.; Kollar B.; Borges T. J.; Eskandari A. S.; Zinter M. A.; Cai S.; Assaker J. P.; Choi J. Y.; Al Dulaijan B. S.; Mansouri A.; Haik Y.; Tannous B. A.; van Son W. J.; Leuvenink H. G. D.; Pomahac B.; Riella L. V.; Tang L.; Seelen M. A. J.; Irvine D. J.; Azzi J. R. Regulatory T cells engineered with TCR signaling–responsive IL-2 nanogels suppress alloimmunity in sites of antigen encounter. Sci. Transl. Med. 2020, 12, eaaw4744 10.1126/scitranslmed.aaw4744. 33177180 PMC8519505

[ref18] Perdue L. A.; Do P.; David C.; Chyong A.; Kellner A. V.; Ruggieri A.; Kim H. R.; Salaita K.; Lesinski G. B.; Porter C. C.; Dreaden E. C. Optical Control of Cytokine Signaling via Bioinspired. Polymer-Induced Latency. Biomacromolecules 2020, 21, 2635–2644. 10.1021/acs.biomac.0c00264. 32374589 PMC8496955

[ref19] Henderson T. A.; Morries L. D. Near-infrared photonic energy penetration: can infrared phototherapy effectively reach the human brain?. Neuropsychiatr. Dis. Treat. 2015, 11, 2191 10.2147/NDT.S78182. 26346298 PMC4552256

[ref20] Gorka A. P.; Nani R. R.; Zhu J.; Mackem S.; Schnermann M. J. A Near-IR Uncaging Strategy Based on Cyanine Photochemistry. J. Am. Chem. Soc. 2014, 136, 14153–14159. 10.1021/ja5065203. 25211609 PMC4195383

[ref21] Padwa A. 1,3-Dipolar cycloaddition chemistry; Wiley-Interscience, 1984.

[ref22] Agard N. J.; Prescher J. A.; Bertozzi C. R. A Strain-Promoted [3 + 2] Azide–Alkyne Cycloaddition for Covalent Modification of Biomolecules in Living Systems. J. Am. Chem. Soc. 2004, 126, 15046–15047. 10.1021/ja044996f. 15547999

[ref23] Devaraj N. K.; Weissleder R.; Hilderbrand S. A. Tetrazine-Based Cycloadditions: Application to Pretargeted Live Cell Imaging. Bioconjugate Chem. 2008, 19, 2297–2299. 10.1021/bc8004446. PMC2677645 19053305

[ref24] Meijer E. F. J.; Jeong H.-S.; Pereira E. R.; Ruggieri T. A.; Blatter C.; Vakoc B. J.; Padera T. P. Murine chronic lymph node window for longitudinal intravital lymph node imaging. Nat. Protoc. 2017, 12, 1513–1520. 10.1038/nprot.2017.045. 28683064 PMC5592697

[ref25] Firl D. J.; Degn S. E.; Padera T.; Carroll M. C. Capturing change in clonal composition amongst single mouse germinal centers. eLife 2018, 7, e33051 10.7554/eLife.33051. 30066671 PMC6070335

[ref26] Wu H.; Estrella V.; Beatty M.; Abrahams D.; El-Kenawi A.; Russell S.; Ibrahim-Hashim A.; Longo D. L.; Reshetnyak Y. K.; Moshnikova A.; Andreev O. A.; Luddy K.; Damaghi M.; Kodumudi K.; Pillai S. R.; Enriquez-Navas P.; Pilon-Thomas S.; Swietach P.; Gillies R. J. T-cells produce acidic niches in lymph nodes to suppress their own effector functions. Nat. Commun. 2020, 11, 4113 10.1038/s41467-020-17756-7. 32807791 PMC7431837

[ref27] Shao Y.; Taniguchi K.; Townshend R. F.; Miki T.; Gumucio D. L.; Fu J. A pluripotent stem cell-based model for post-implantation human amniotic sac development. Nat. Commun. 2017, 8, 208 10.1038/s41467-017-00236-w. 28785084 PMC5547056

[ref28] Yu L.; Wei Y.; Duan J.; Schmitz D. A.; Sakurai M.; Wang L.; Wang K.; Zhao S.; Hon G. C.; Wu J. Blastocyst-like structures generated from human pluripotent stem cells. Nature 2021, 591, 620–626. 10.1038/s41586-021-03356-y. 33731924

[ref29] Hunter R.; Imbach K. J.; Zhou C.; Dougan J.; Hamilton J. A. G.; Chen K. Z.; Do P.; Townsel A.; Gibson G.; Dreaden E. C.; Waller E. K.; Haynes K. A.; Henry C. J.; Porter C. C. B-cell acute lymphoblastic leukemia promotes an immune suppressive microenvironment that can be overcome by IL-12. Sci. Rep. 2022, 12, 11870 10.1038/s41598-022-16152-z. 35831470 PMC9279427

[ref30] Do P.; Perdue L. A.; Chyong A.; Hunter R.; Dougan J.; Henry C. J.; Porter C. C.; Dreaden E. C. Rapid Assembly and Screening of Multivalent Immune Cell-Redirecting Therapies for Leukemia. ACS Comb. Sci. 2020, 22, 533–541. 10.1021/acscombsci.0c00081. 32786324 PMC8496977

[ref31] Wigginton J. M.; Komschlies K. L.; Back T. C.; Franco J. L.; Brunda M. J.; Wiltrout R. H. Administration of Interleukin 12 With Pulse Interleukin 2 and the Rapid and Complete Eradication of Murine Renal Carcinoma. J. Natl. Cancer Inst. 1996, 88, 38–43. 10.1093/jnci/88.1.38. 8847724

[ref32] Zaki M. H.; Wysocka M.; Everetts S. E.; Rook A. H.; Wang K. S.; French L. E.; Ritz J. Synergistic Enhancement of Cell-Mediated Immunity by Interleukin-12 Plus Interleukin-2: Basis for Therapy of Cutaneous T Cell Lymphoma. J. Invest. Dermatol. 2002, 118, 366–371. 10.1046/j.1523-1747.2002.01646.x. 11841558

[ref33] Lisiero D. N.; Soto H.; Liau L. M.; Prins R. M. Enhanced Sensitivity to IL-2 Signaling Regulates the Clinical Responsiveness of IL-12–Primed CD8<sup>+</sup> T Cells in a Melanoma Model. J. Immunol. 2011, 186, 5068–5077. 10.4049/jimmunol.1003317. 21430221 PMC3532507

[ref34] Bonis-O’Donnell J. T. D.; Page R. H.; Beyene A. G.; Tindall E. G.; McFarlane I. R.; Landry M. P. Dual Near-Infrared Two-Photon Microscopy for Deep-Tissue Dopamine Nanosensor Imaging. Adv. Funct. Mater. 2017, 27, 1702112 10.1002/adfm.201702112.

[ref35] Mullard A. Restoring IL-2 to its cancer immunotherapy glory. Nat. Rev. Drug Discovery 2021, 20, 163–165. 10.1038/d41573-021-00034-6. 33603157

[ref36] Lopes J. E.; Fisher J. L.; Flick H. L.; Wang C.; Sun L.; Ernstoff M. S.; Alvarez J. C.; Losey H. C. ALKS 4230: a novel engineered IL-2 fusion protein with an improved cellular selectivity profile for cancer immunotherapy. J. Immunother. Cancer 2020, 8, e000673 10.1136/jitc-2020-000673. 32317293 PMC7204809

[ref37] Goebeler M.-E.; Bargou R. C. T cell-engaging therapies—BiTEs and beyond. Nat. Rev. Clin. Oncol. 2020, 17, 418–434. 10.1038/s41571-020-0347-5. 32242094

[ref38] Suurs F. V.; Lub-de Hooge M. N.; de Vries E. G. E.; de Groot D. J. A. A review of bispecific antibodies and antibody constructs in oncology and clinical challenges. Pharmacol. Ther. 2019, 201, 103–119. 10.1016/j.pharmthera.2019.04.006. 31028837

[ref39] Kim C. K.; Adhikari A.; Deisseroth K. Integration of optogenetics with complementary methodologies in systems neuroscience. Nat. Rev. Neurosci. 2017, 18, 222–235. 10.1038/nrn.2017.15. 28303019 PMC5708544

[ref40] Levskaya A.; Weiner O. D.; Lim W. A.; Voigt C. A. Spatiotemporal control of cell signalling using a light-switchable protein interaction. Nature 2009, 461, 997–1001. 10.1038/nature08446. 19749742 PMC2989900

[ref41] Boyman O.; Sprent J. The role of interleukin-2 during homeostasis and activation of the immune system. Nat. Rev. Immunol. 2012, 12, 180–190. 10.1038/nri3156. 22343569

[ref42] Abbas A. K.; Trotta E.; Simeonov D. R.; Marson A.; Bluestone J. A. Revisiting IL-2: Biology and therapeutic prospects. Sci. Immunol. 2018, 3, eaat1482 10.1126/sciimmunol.aat1482. 29980618

[ref43] Chen B.-M.; Cheng T.-L.; Roffler S. R. Polyethylene Glycol Immunogenicity: Theoretical, Clinical, and Practical Aspects of Anti-Polyethylene Glycol Antibodies. ACS Nano 2021, 15, 14022–14048. 10.1021/acsnano.1c05922. 34469112

[ref44] Sebestyen Z.; Prinz I.; Déchanet-Merville J.; Silva-Santos B.; Kuball J. Translating gammadelta (γδ) T cells and their receptors into cancer cell therapies. Nat. Rev. Drug Discovery 2020, 19, 169–184. 10.1038/s41573-019-0038-z. 31492944

[ref45] Laskowski T. J.; Biederstädt A.; Rezvani K. Natural killer cells in antitumour adoptive cell immunotherapy. Nat. Rev. Cancer 2022, 22, 557–575. 10.1038/s41568-022-00491-0. 35879429 PMC9309992

[ref46] Myers J. A.; Miller J. S. Exploring the NK cell platform for cancer immunotherapy. Nat. Rev. Clin. Oncol. 2021, 18, 85–100. 10.1038/s41571-020-0426-7. 32934330 PMC8316981

[ref47] Hansen M. J.; Velema W. A.; Lerch M. M.; Szymanski W.; Feringa B. L. Wavelength-selective cleavage of photoprotecting groups: strategies and applications in dynamic systems. Chem. Soc. Rev. 2015, 44, 3358–3377. 10.1039/C5CS00118H. 25917924

[ref48] Peterson J. A.; Wijesooriya C.; Gehrmann E. J.; Mahoney K. M.; Goswami P. P.; Albright T. R.; Syed A.; Dutton A. S.; Smith E. A.; Winter A. H. Family of BODIPY Photocages Cleaved by Single Photons of Visible/Near-Infrared Light. J. Am. Chem. Soc. 2018, 140, 7343–7346. 10.1021/jacs.8b04040. 29775298

[ref49] Bardhan A.; Deiters A. Development of photolabile protecting groups and their application to the optochemical control of cell signaling. Curr. Opiin. Struct. Biol. 2019, 57, 164–175. 10.1016/j.sbi.2019.03.028. PMC7026702 31132552

